# Cancer-associated Fibroblasts Confer Osimertinib Resistance in Non-small Cell Lung Cancer Cells via NRG1-mediated HER3/AKT Signaling

**DOI:** 10.7150/jca.111383

**Published:** 2025-08-11

**Authors:** Sijia Zheng, Limin Cao, Jiayi Zhang, Qicheng Zhang, Yinghui Ren, Min Wang, Yongmei Qian, Bingbing Li, Xiang Wu, Zhaowei Meng, Ke Xu

**Affiliations:** 1 Tianjin Key Laboratory of Lung Cancer Metastasis and Tumor Microenvironment, Tianjin Lung Cancer Institute, Tianjin Medical University General Hospital, Tianjin 300052, China.; 2 Department of Lung Cancer Surgery, Tianjin Medical University General Hospital, Tianjin 300052, China.; 3 Department of Clinical Lab, Tianjin Children's Hospital, Tianjin 300134, China.; 4 Department of Anesthesiology, Tianjin First Central Hospital, Tianjin 300192, China.; 5 Core Facility Center, Tianjin Medical University General Hospital, Tianjin 300052, China.; 6 Department of Nuclear Medicine, Tianjin Medical University General Hospital, Tianjin 300052, China.

**Keywords:** Cancer-associated fibroblasts, Lung cancer, Osimertinib resistance, NRG1, HER3

## Abstract

Osimertinib is a third-generation epidermal growth factor receptor (EGFR) tyrosine kinase inhibitor (TKI); it has achieved favorable progression-free survival (PFS) in non-small cell lung cancer (NSCLC) patients with EGFR mutation, however, the resistance occurs in most patients, and the underlying mechanism remain to be elucidated. Cancer-associated fibroblasts (CAFs) are major stromal cells in tumor microenvironment. Despite accumulating evidence suggests that CAFs contribute to drug resistance, the role of CAFs in osimertinib resistance in NSCLC is not fully understood. Here, we reported that CAFs promoted the resistance of NSCLC cells to osimertinib through enhancing stemness of NSCLC cells and reducing apoptosis induced by osimertinib. CAFs possessed a high level of Neuregulin-1 (NRG1), and CAFs-secreted NRG1 mediated the promoting effect of CAFs on osimertinib resistance, demonstrated by applying recombinant human NRG1 (rhNRG1) and NRG1 knockdown. We also found that osimertinib stimulated NRG1 secretion by CAFs, which may further enhance osimertinib resistance. Further study revealed that CAFs promoted the resistance of NSCLC cells to osimertinib via NRG1-mediated HER3/AKT/NF-κB pathway. Moreover, the mouse xenograft study demonstrated that CAFs enhanced osimertinib-treated tumor growth *in vivo*. Our finding highlights the potential value of CAFs-derived NRG1 as a novel therapeutic target for osimertinib resistance in lung cancer.

## Introduction

Lung cancer is the most common and fatal malignancy worldwide; there are nearly 2.5 million new case each year 1. The major subtype of lung cancer is NSCLC, it accounts for approximate 85% of total lung cancer 2. Despite the treatment of lung cancer has been greatly improved, the 5-year survival rate is below 20% 1, highlighting the need of novel therapeutic strategies for lung cancer.

EGFR is a key driver oncogene in NSCLC. Numerous studies have shown that targeted therapy with EGFR-TKIs has achieved favorable PFS in NSCLC patients with EGFR mutation (in particular, exon 19 deletion or exon 21 L858R), however, acquired resistance occurs in most patients after 9-12 months treatment. One of the main causes of the acquired resistance is the secondary mutation T790M in EGFR, which accounts for 50%-60% of EGFR mutation cases 3, 4. Osimertinib is a third-generation irreversible EGFR-TKI, it selectively inhibits EGFR T790M mutation as well as sensitive mutations. Despite osimertinib treatment obtains more than 10 months PFS of patients, the acquired resistance to osimertinib is inevitable. There are several mechanisms underlying osimertinib resistance, including EGFR C797X mutation, amplification in MET, rearrangement in RET, downstream signaling pathways activation, histological transformation, epithelial - mesenchymal transition (MET) 5. The mechanisms are still not fully understood, warranting the exploration of novel mechanisms.

Tumor progression depends on the communication between tumor cells and tumor microenvironment (TME). Among various cellular and non-cellular components in TME, CAFs are major stromal cells. CAFs promote tumor development via secreting growth factors and cytokines 6. In particular, CAFs facilitate drug resistance in a variety of cancers. CAFs promote cisplatin resistance of gastric cancer via SULF1 secretion and TGFBR3-mediated TGF-β signaling pathway 7. CAFs enhance gemcitabine resistance via HIF-1α/miR-21 axis in pancreatic cancer 8. In respect to EGFR-TKI resistance, CAFs promote EMT and gefitinib resistance of NSCLC cells via HGF/IGF-1/ANXA2 signaling 9. Our previous study also demonstrated that CAFs contribute to cisplatin resistance via modulating ANXA3 in lung cancer cells 10.

NRG1 belongs to the NRG gene family, which consists of NRG type I to type VI. Initially NRG1 is identified in nascent ventricular endocardium 11. NRG1 involves in cell proliferation, differentiation, survival, influencing organogenesis and cell differentiation 12. Recent studies reveal that NRG1 play a pivotal role in tumorigenesis and tumor progression. NRG1 is up**-**regulated in several types of cancer including breast cancer, lung cancer, pancreatic cancer, and over**-**expression of NRG1 may activate downstream signaling pathways such as ERBB3/ERBB2 pathway, leading to the stimulation on cancer cell proliferation, invasion, and drug resistance 13. In the present study, we investigated the role of lung cancer-derived CAFs in osimertinib resistance of NSCLC cells. We found that CAFs facilitate osimertinib resistance in NSCLC cells, and NRG1 secreted by CAFs mediates the resistance-promoting effect.

## Materials and Methods

### Reagents and antibodies

Osimertinib was purchased from MCE (Shanghai, China). The recombinant human NRG1 was obtained from Peprotech (Cranbury, NJ). Antibodies against SOX2 (#3579), Nanog (#4903), Survivin (#2808), Caspase-9 (#9508), Cleaved caspase-9 (#20750), NRG1 (#2573S), HER3 (#4757), p-HER3 (#4791), AKT (#4691), p-AKT (#4060), p65 (#4764), p-p65 (#3033) (1:1000 dilution) were purchased from Cell Signaling Technology (Beverly, MA). The anti-OCT4 (#11263-1-AP, 1:1000 dilution) was purchased from Proteintech (Rosemont, IL); anti-Bcl-2 (#WL01556, 1:1000 dilution) was purchased from Wanleibio (Shenyang, China); anti-β-actin (#A2228, 1:5000 dilution) was purchased from Sigma-Aldrich (St. Louis, MO), anti-mouse IgG (#ZB2305, 1:8000 dilution) and goat anti-rabbit (#ZB-2301, 1:8000 dilution) were purchased from ZSGB-BIO (Beijing, China). HER3 inhibitor TX1-85-1 was purchased from MCE (Shanghai, China). AKT inhibitor perifosine and NF-κB inhibitor JSH23 were purchased from TargetMol (Shanghai, China).

### Cell culture and lung stromal fibroblasts

Human NSCLC cell lines H1975, PC9, and human lung bronchial epithelial cell line BEAS-2B cells were obtained from the American Type Culture Collection (Manassas, VA). PC9 and BEAS-2B cells were cultured in DMEM medium, and H1975 cells were cultured in RPMI-1640 medium. The medium was supplemented with 10% fetal bovine serum (GIBCO; Grand Island, NY); and cells were cultured at 37°C, 5% CO_2_. Human CAFs and normal fibroblasts (NFs) were isolated from tumor and adjacent non-tumor tissues of NSCLC patients, respectively. CAFs and NFs were characterized and conditioned medium (CM) was collected as previously described 14. CAFs and NFs were grown and used within 10 passages. The patients received surgery at Tianjin Medical University General Hospital (TMUGH, Tianjin, China), and the informed consent was gained from the patients. The study obtained the permission from the institutional Ethical Review Committee of TMUGH.

### Cell proliferation assay

NSCLC cells were plated in 96-well plates at a density of 4×10^3^ cells/well, then treated with Osimertinib at different concentrations. Cell viability was examined by the Cell Counting Kit-8 (CCK-8, Dojindo, Kumamoto, Japan) following the manufacturer's instructions. The median inhibitory concentration IC_50_ values were calculated using GraphPad Prism 8.0 software (La Jolla, CA).

### Colony formation assay

Cells were plated in 6-well plates at 1000 cells/well, and cultured for 10 - 14 days successively. Colonies were stained by 1% crystal violet. Images were collected by a scanner (Canon, Tokyo, Japan) and the colonies were counted.

### Sphere formation assay

Cells were plated in ultra-low attachment plates (Corning, New York, NY) in DMEM medium supplied with 20 ng/ml epidermal growth factor, 20 ng/ml basic fibroblast growth factor, and 2% B27 (GIBCO; Grand Island, NY). After 10 days, spheres were observed and counted under a microcopy.

### Apoptosis assay

Cells were treated with Osimertinib for 48 h. After treatment, the apoptotic cells were determined using an Annexin V-FITC Apoptosis Detection Kit (Solarbio, Beijing, China), following the manufacturer's instructions. Briefly, cells were washed with PBS and re-suspended in binding buffer. Annexin V-FITC and PI were then added to the cells, and cells were incubated for 5 min in the dark. Apoptosis was detected on a FACSAria flow cytometer (Becton Dickenson, San Jose, CA).

### Enzyme-linked immunosorbent assay (ELISA)

The NRG1 levels in culture medium were detected by ELISA assay as described previously 15. Briefly, medium was collected, and the levels of NRG1 in medium were detected by an ELISA assay kit (Lanpaibio, Shanghai, China). The concentrations were presented as pg/mL.

### RNA interference and transfection

CAFs were transfected with siNRG1 or siControl (GenePharma, Shanghai, China) using Lipofectamine 3000 (Thermo Fisher Scientific, Waltham, MA). The sequences of siRNA duplex for NRG1 were: sense 5'-GGCUGAUUCUGGAGAGUAU-3', antisense 5'-AUACUCUCCAGAAUCAGCC-3'; for control were, sense 5'-UUCUCCGAACGUGUCACGUTT-3', antisense 5'-ACGUGACACGUUCGGAGAATT-3'.

### Quantitative PCR (qPCR)

Gene expression was analyzed by qPCR as previously described 16. RNA was extracted from cells using TRIzol (Invitrogen, Carlsbad, CA), and reverse transcription was performed using the Takara kit (Dalian, China). qPCR was carried out using the Power SYBR Green Master Mix (Thermo Fisher Scientific) on an ABI 7500HT Sequence Detector. GAPDH was used for normalization. The primer sequences for NRG1 were: forward AGTCCTTCGGTGTGAAACCAG, reverse TGCGAAGTTCTGACTTCCCTG; for GAPDH were: forward TGCACCACCAACTGCTTAGC; reverse GGCATGGACTGTGGTCATGAG.

### Western blotting

Cell lysates were extracted using a RIPA lysis buffer (Sigma-Aldrich, St Louis, MO). Proteins were separated by SDS-PAGE and transferred onto a nitrocellulose membrane (Millipore, Bedford, MA). After blocking with 5% non-fat milk for 1h, the membranes were probed with primary antibodies and the corresponding HRP-conjugated secondary antibodies. Finally, the protein bands were visualized using the ECL Western Blotting System (Thermo Fisher Scientific) following the manufacturer's instructions.

### Xenograft mouse model

Five-week old BALB/c nude mice were purchased from the Cancer Institute of Chinese Academy of Medical Science (Beijing, China). The xenograft experiments were approved by the Tianjin Medical University Institutional Animal Care and Use Committee. Mice were randomly allocated into four groups (n = 7/group). To investigate the effect of CAFs on lung cancer cells growth* in vivo*, 1×10^6^ H1975 cells or 1×10^6^ H1975 cells mixed with 3×10^6^ CAFs (ratio1:3) were re-suspended in 100 μL PBS and injected subcutaneously into the mice. When tumors were formed (after 7 days), mice were treated with Osimertinib (2.5 mg/kg/d). The tumor growth was monitored, and tumor volume was calculated as: volume = d^2^ × D/2, (d and D were the shortest and the longest diameters). The experiment was terminated after 5 weeks, and tumor tissues were removed for further examinations.

### Statistical analysis

The data analysis was performed with GraphPad Prism 9 (GraphPad Software, San Diego, CA). For comparisons of 2 groups, Student's *t*-test was performed; for comparisons of multiple groups, one-way ANOVA was performed. All experiments were performed for at least three times. Data were presented as mean ± standard deviation. The *p* value < 0.05 was considered to be statistically significant.

## Results

### CAFs enhance the resistance of NSCLC cells to osimertinib

Increasing evidence supports that CAFs mediate the drug resistance of cancer cells. Our previous study showed that CAFs contribute to cisplatin resistance via modulating ANXA3 in NSCLC cells 10, however, the role of CAFs in the resistance of NSCLC cell to osimertinib remains largely unknown. In the present study, we established CAFs and NFs cell lines from cancerous and adjacent non-cancerous specimens of NSCLC patients. We also selected EGFR-mutant NSCLC cell lines PC9 (EGFR del 19) and H1975 (L858R/T790M) to assess the effect of osimertinib.

**Figure 1:**
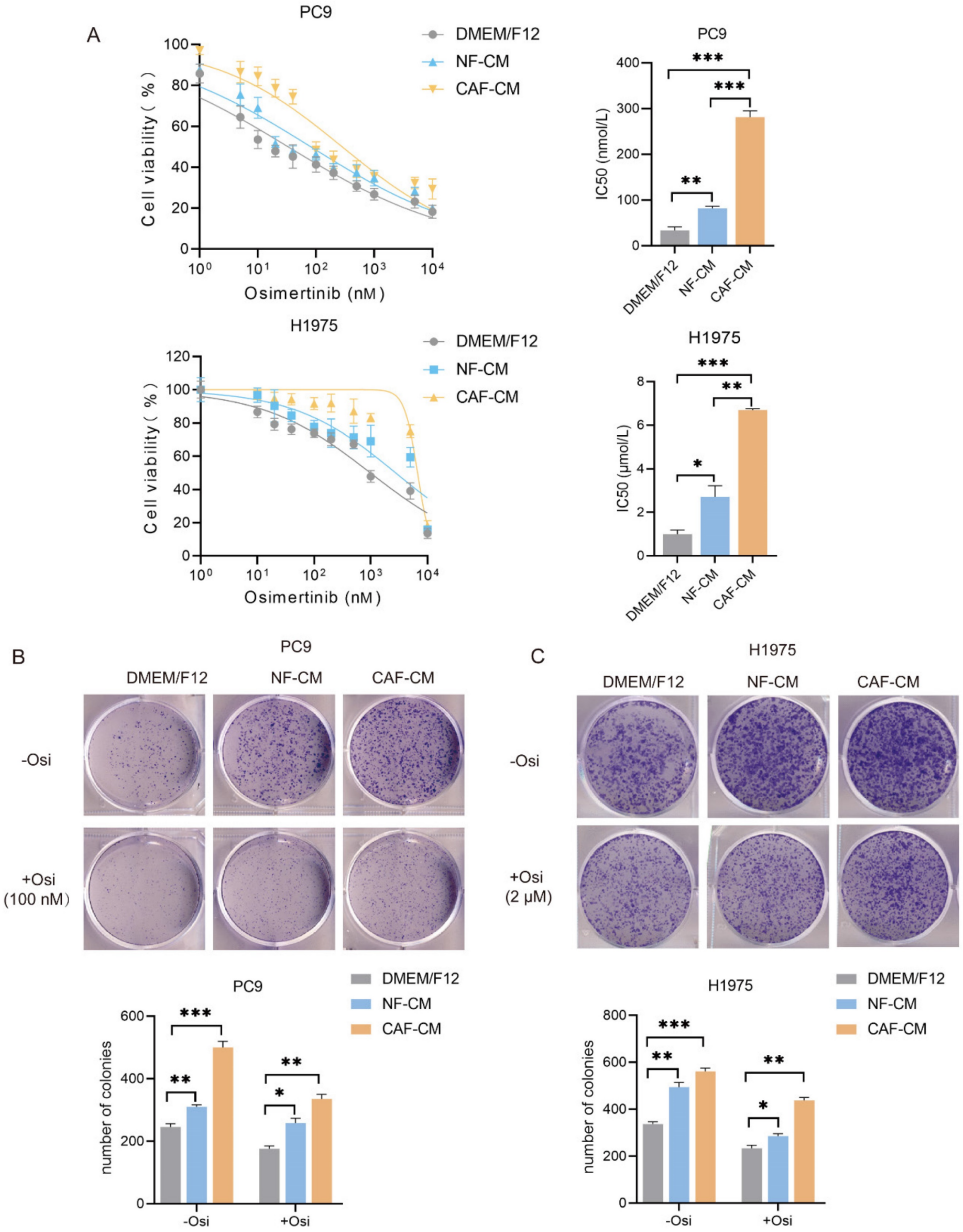

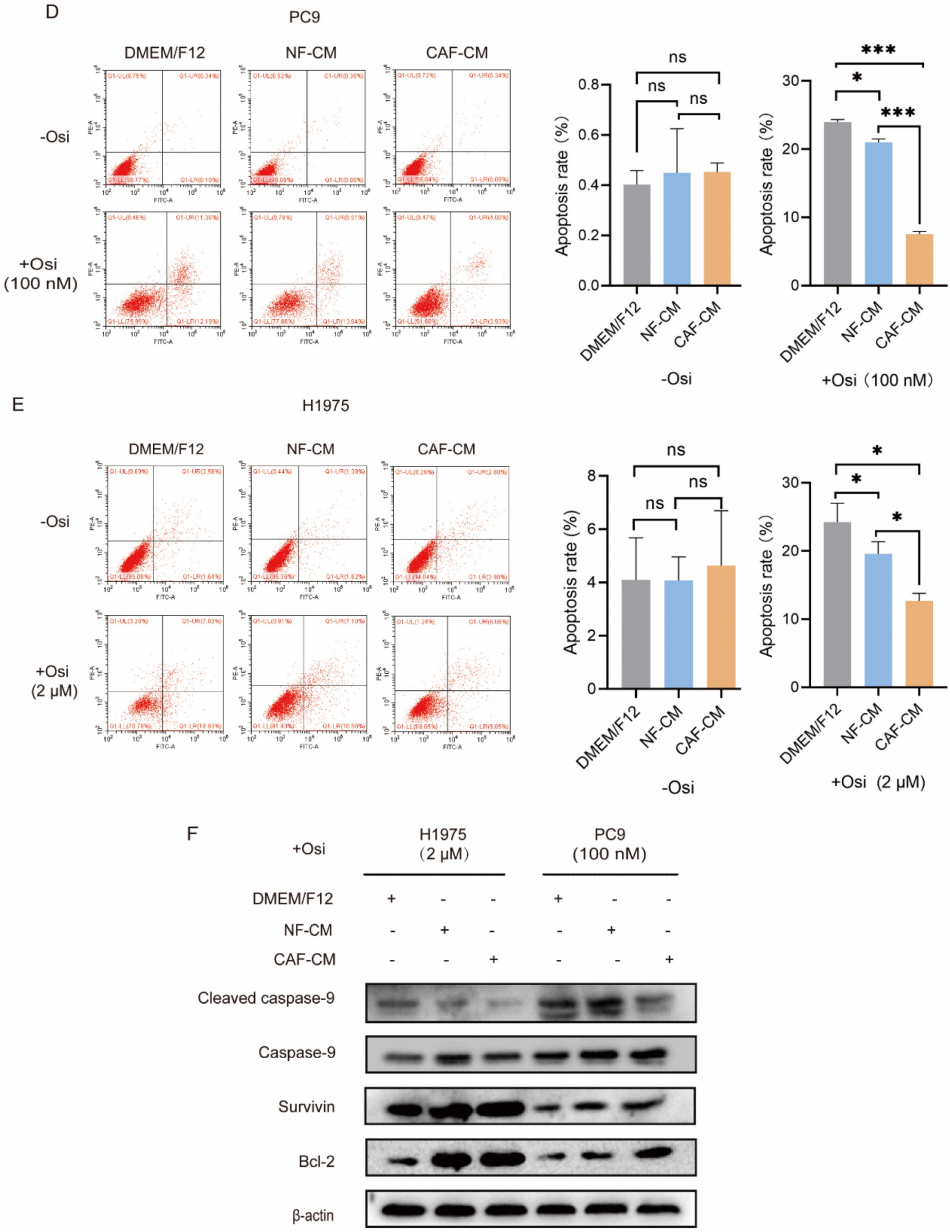

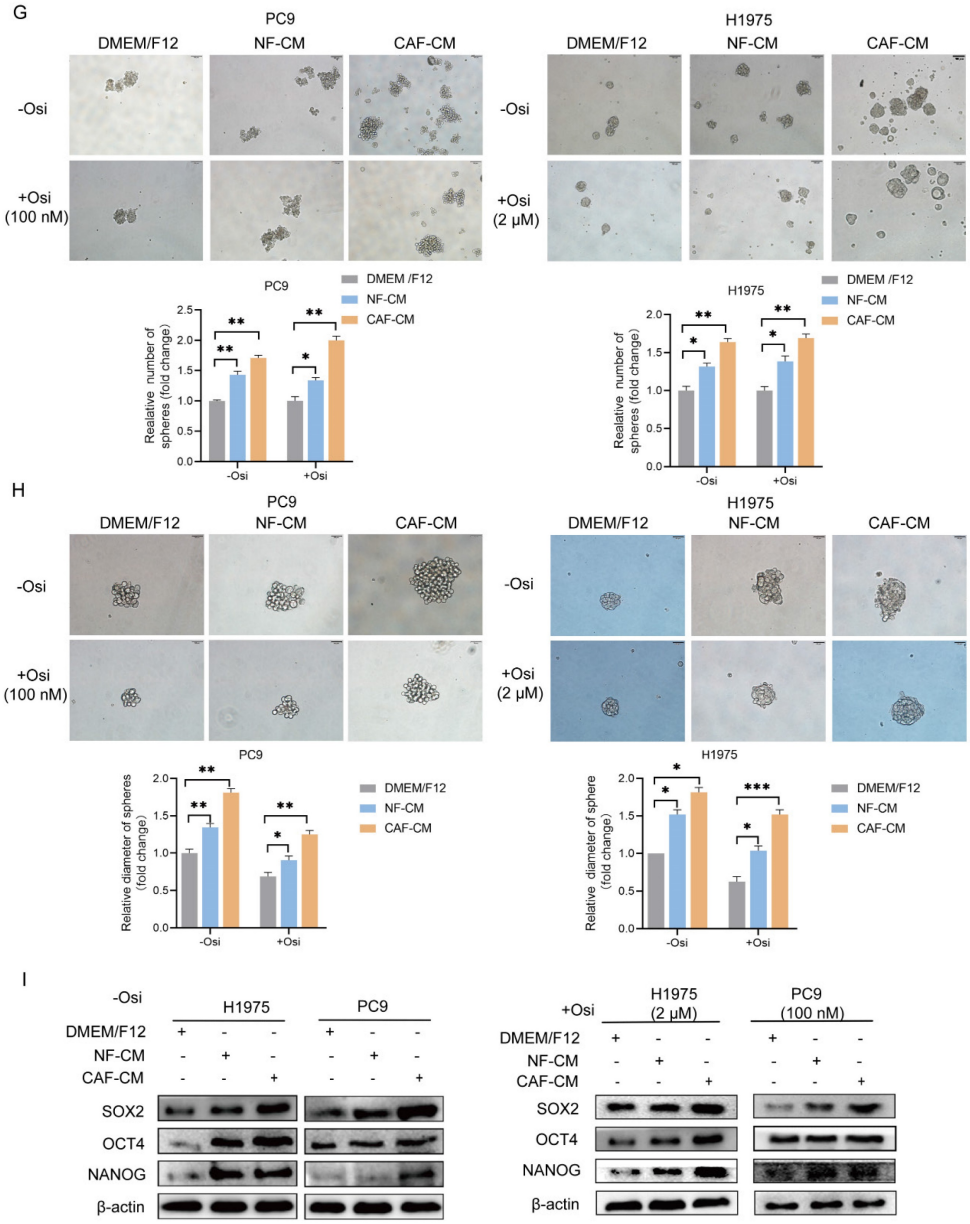
** CAFs enhance the resistance of NSCLC cell to osimertinib.** NSCLC cells were cultured in DMEM/F12, NF-CM or CAF-CM. (**A**) Cells were treated with osimertinib (0 - 10 μM) for 48 h. Cell viability was detected by CCK-8 kit. (**B, C**) Cells were treated with osimertinib (100 nM or 2 μM). The colony formation assay was performed after 1-2 weeks. (**D, E**) Cells were treated with osimertinib (100 nM or 2 μM) for 48 h. The apoptosis level was detected by flow cytometry. (**F**) Cells were treated with osimertinib (100 nM or 2 μM). The protein expressions were detected by Western blotting for 48 h. (**G, H**) Cells were treated with osimertinib (100 nM or 2 μM). Cell sphere formation experiment was performed after 1-2 weeks. G: scale bar = 100 μm. H: scale bar = 50 μm. (**I**) Cells were treated with osimertinib (100 nM or 2 μM) for 48 h. The protein expressions were detected by Western blotting. Data represented the mean ± SD from three independent experiments. Columns, mean; bars, SD. *P<0.05, **P<0.01, ***P<0.001.

To investigate the effect of CAFs on the sensitivity of NSCLC cells to osimertinib, H1975 and PC9 cells were incubated with CAF-CM or NF-CM, and treated with osimertinib. CCK-8 assay showed that both CAFs and NFs enhanced the resistance of NSCLC cells to osimertinib, and CAFs had a stronger effect than NFs. The IC_50_ value of osimertinib on PC9 cells were increased from 33.1 nmol/L (DMEM/F12) and 81.7 nmol/L (NF-CM) to 281.2 μmol/L (CAF-CM). For H1975 cells, IC_50_ value ranged from 0.98 μmol/L (DMEM/F12), 2.76 μmol/L (NF-CM) to 6.70 μmol/L (CAF-CM) (Figure 1A). For the ability of colony formation, CAFs enhanced colony formation in both untreated and osimertinib-treated cells (Figure 1B, C).

To further evaluate the effect of CAFs on osimertinib resistance, the apoptosis rate of lung cancer cells was detected by flow cytometry. In the absence of osimertinib, there was no significant difference in apoptosis rate among all groups, however, in osimertinib treatment groups, both CAFs and NFs reduced apoptosis levels in NSCLC cells, and CAFs were more effective (Figure 1D, E). The Western blot results supported this finding by showing that caspase-9 activation was attenuated by CAFs, meanwhile Survivin and anti-apoptosis gene Bcl-2 were upregulated (Figure 1F). Given that the stemness of cancer cells is responsible for drug resistance 17, we next evaluated the effect of CAFs on NSCLC cell stemness. Sphere formations were assessed by examining sphere number and size. Figure 1G and H showed that both CAFs and NFs facilitated the sphere formation of lung cancer cells, and the effect of CAFs was stronger. Notably, when we compared the sphere size of untreated- and osimertinib-treated group, we found that osimertinib reduced the size of sphere in DMEM/F12, NF-CM and CAF-CM group, respectively (Figure H). Moreover, the increased expression of stemness-related genes SOX2, NANOG and OCT4 by CAFs further demonstrated that CAFs promoted the stemness of NSCLC cells (Figure 1I). Collectively, these results indicated that CAFs enhances the resistance of NSCLC cells to osimertinib through facilitating stemness and reducing apoptosis.

### CAFs possess a high level of NRG1

**Figure 2:**
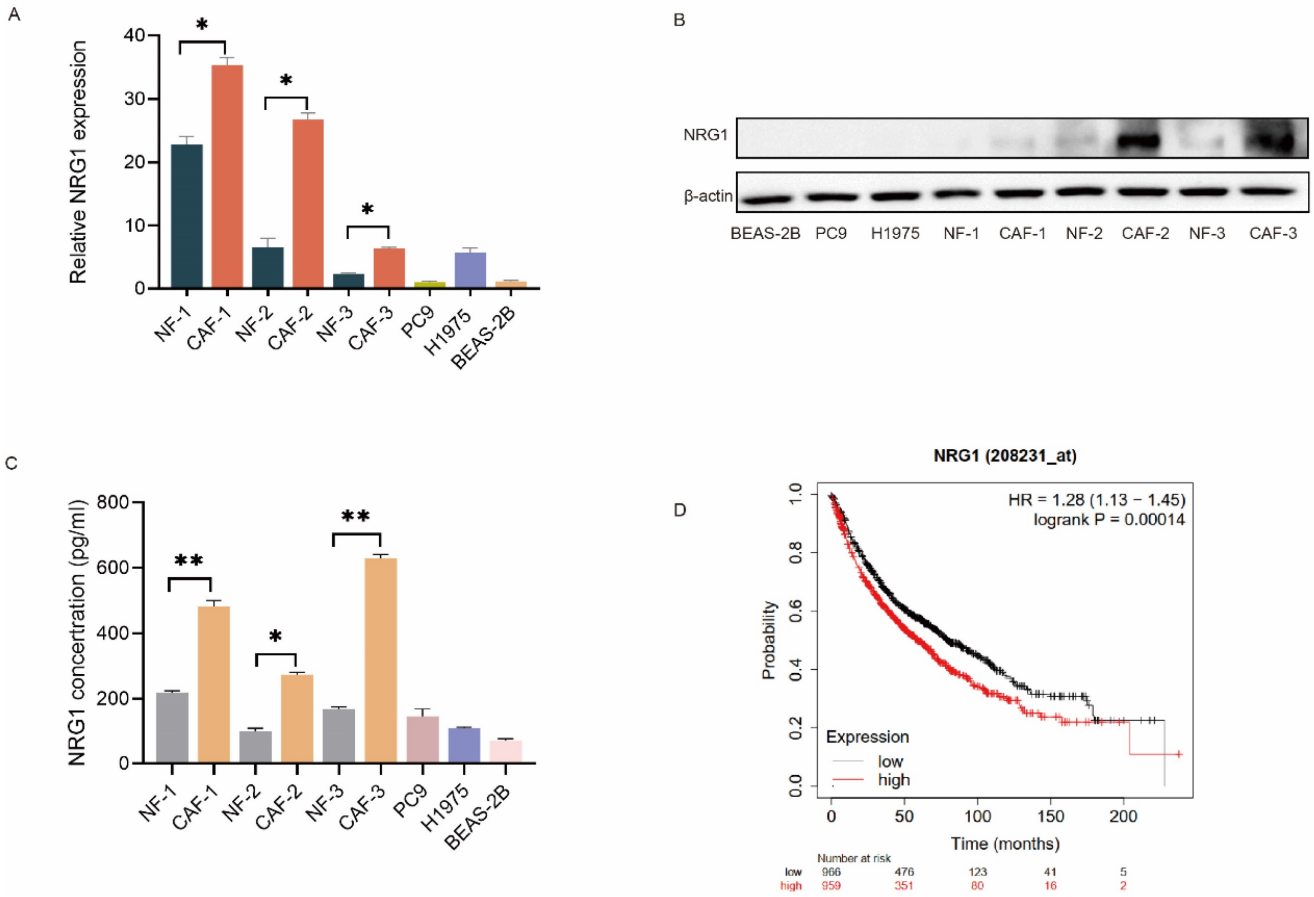
** CAFs possess a high level of NRG1.** (**A**) The mRNA level of NRG1 in paired NFs and CAFs (n = 3), lung cancer cells, and normal lung bronchial epithelial cells were detected by qPCR. (**B**) The protein expressions of NRG1 in paired NFs and CAFs (n = 3), lung cancer cells, and normal lung bronchial epithelial cells were detected by Western blotting. (**C**) The secreted NRG1 by paired NFs and CAFs (n = 3), lung cancer cells, and normal lung bronchial epithelial cells were detected by ELISA kit. (**D**) Kaplan-Meier curve show the association between NRG1 expression and survival of lung cancer patients. Columns, mean; bars, SD. **p < 0.01, ***p < 0.001.

Since CAFs interact with tumor cells via secreting cytokines and growth factors, we then explored the growth factors which mediate CAFs' effect. We investigated the differentially expressed genes between CAFs and NFs by RNA sequencing in our previous study 10, we found that NRG1 is highly expressed in CAFs compared to NFs. We then compared the expression of NRG1 among fibroblasts, lung cancer cells and normal lung bronchial epithelial cells. qPCR results showed that NRG1 level in fibroblasts is higher than that in lung cancer cells and normal lung bronchial epithelial cells, and CAFs possess higher level than NFs (Figure 2A). The Western blot results further confirmed this finding (Figure 2B).

Next, we examined the NRG1 secretion from CAFs by ELISA assay. Figure 2C revealed that NRG1 level in CAF-CM was higher than that in NF-CM. Notably, both CAFs and NFs released more NRG1 than lung cancer cells and normal lung bronchial epithelial cells. Furthermore, we assessed the clinical significance of NRG1 in lung cancer. Kaplan-Meier analysis of survival indicated that high level of NRG1 was significantly associated with poor survival rate of lung cancer patients (Figure 2D). Taken together, these data suggested NRG1 as a good candidate for mediating CAFs' effect on osimertinib resistance of NSCLC cells.

### Osimertinib stimulates NRG1 secretion from CAFs

**Figure 3:**
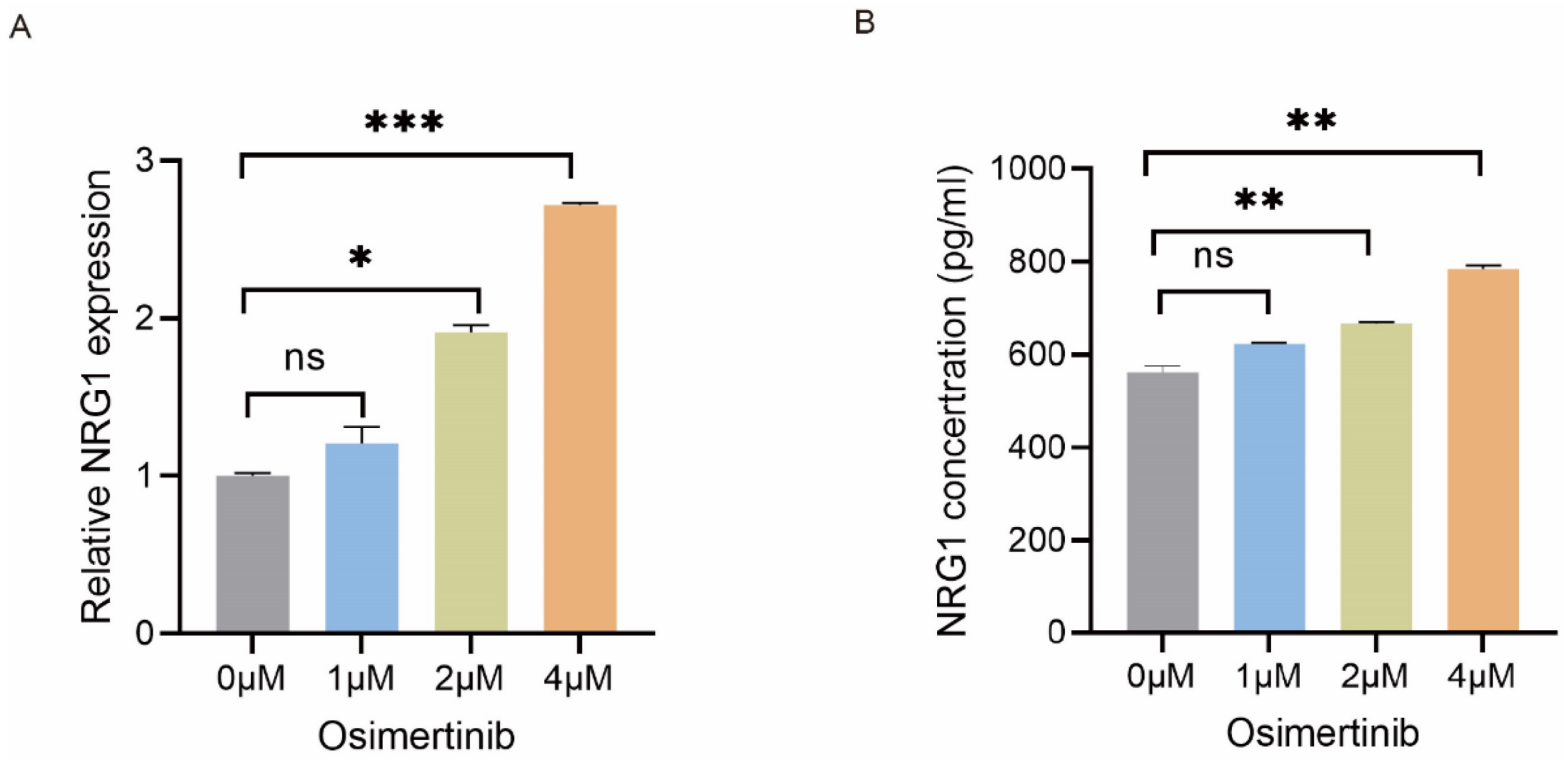
** Osimertinib stimulates NRG1 secretion from CAFs.** CAFs were treated with different concentrations of osimertinib. (**A**) The mRNA level of NRG1 was detected by q-PCR after 24 h. (**B**) NRG1 level in medium was detected by ELISA after 48 h. Columns, mean; bars, SD. **p < 0.01, ***p < 0.001.

To investigate the effect of osimertinib on NRG1 secretion from CAFs, CAFs were treated with 0 - 4 µM of osimertinib for 48 h, then NRG1 mRNA expression in CAFs was detected by qPCR. Figure 3A showed that osimertinib increased NRG1 mRNA expression in CAFs. Consistent with this finding, the NRG1 level in CAF-CM was also elevated (Figure 3B). This data implicated that while CAFs possess high level of NRG1, osimertinib further stimulates the release of NRG1 from CAFs, which may play a crucial role in CAFs' effect on osimertinib resistance.

### NRG1 mediates the promoting effect of CAFs on NSCLC cells growth

**Figure 4:**
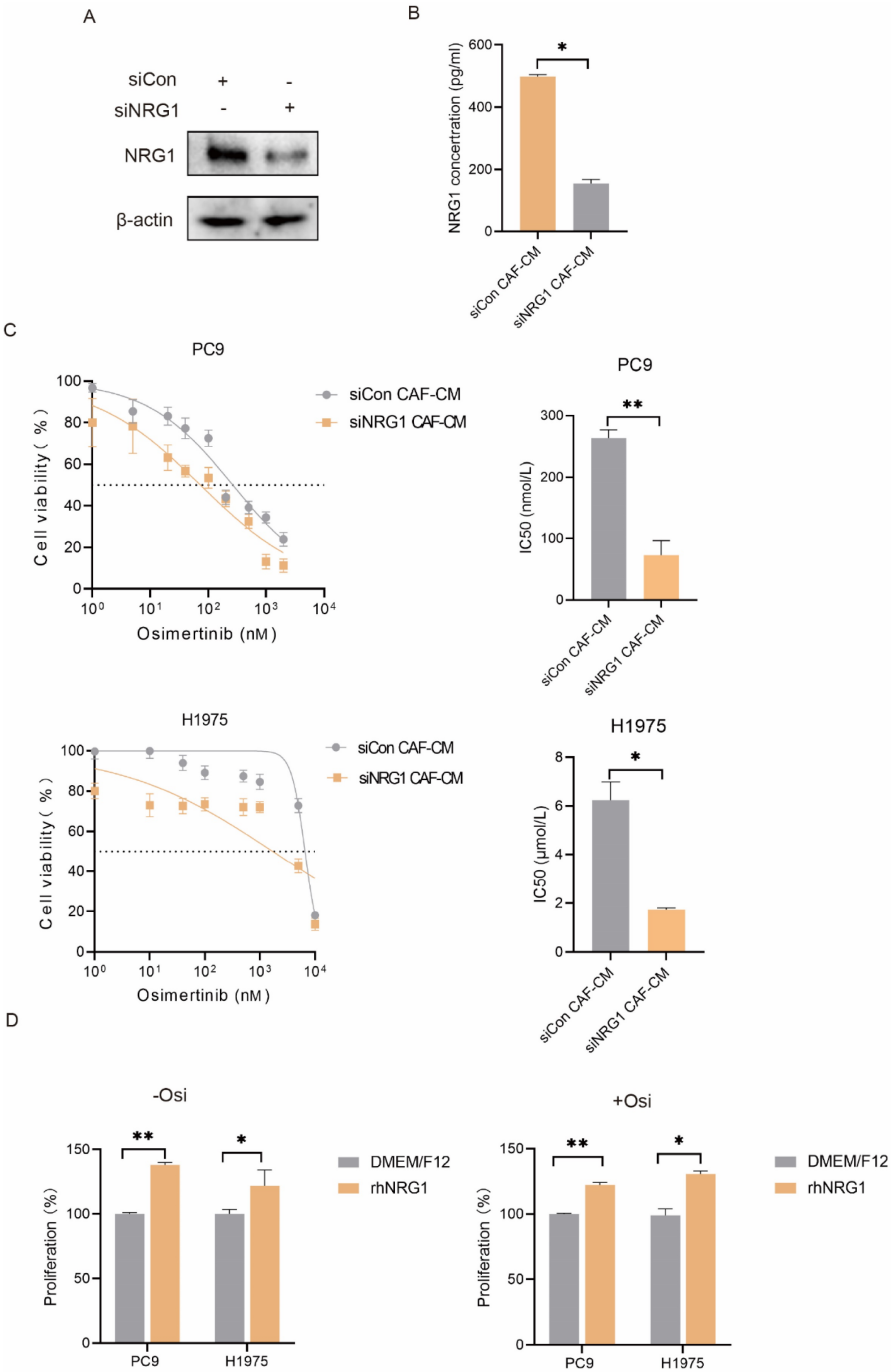
** NRG1 mediates the promoting effect of CAFs on NSCLC cell growth.** (**A, B**) NRG1 was knocked down in CAFs, and NRG1 level in CAF-CM was detected by ELISA. (**C**) Lung cancer cells were cultured in CAF-CM and treated with osimertinib for 48 h. Cell viability was detected by CCK-8 kit. (**D**) rhNRG1 (100 ng/mL) was added to culture medium. Cell were treated with osimertinib, and cell viability was detected by CCK-8 after 48 h. Columns, mean; bars, SD. **p < 0.01, ***p < 0.001.

NRG1 is a secretory protein and is reported to be involved in tumor growth, metastasis and drug resistance 13. This drove us to evaluate the potential role of CAF-secreted NRG1 in osimertinib resistance. The NRG1 level in culture medium was manipulated by different approaches. We first knocked down NRG1 in CAFs, and found that CAF-secreted NRG1 was dramatically reduced (Figure 4A, B). Then CAF-CM was collected and used to culture lung cancer cells. As shown in Figure 4C, knockdown of NRG1 in CAFs significantly mitigated the promoting effect of CAFs on lung cancer cell growth. We further added rhNRG1 to the culture medium, and Figure 4D revealed that NRG1 enhanced the proliferation of lung cancer cell, in both untreated- and osimertinib-treated group. Altogether, these results demonstrated that CAFs enhance NSCLC cell growth via NRG1 secretion.

### NRG1 mediates the effect of CAFs on resistance of NSCLC cells to osimertinib

**Figure 5:**
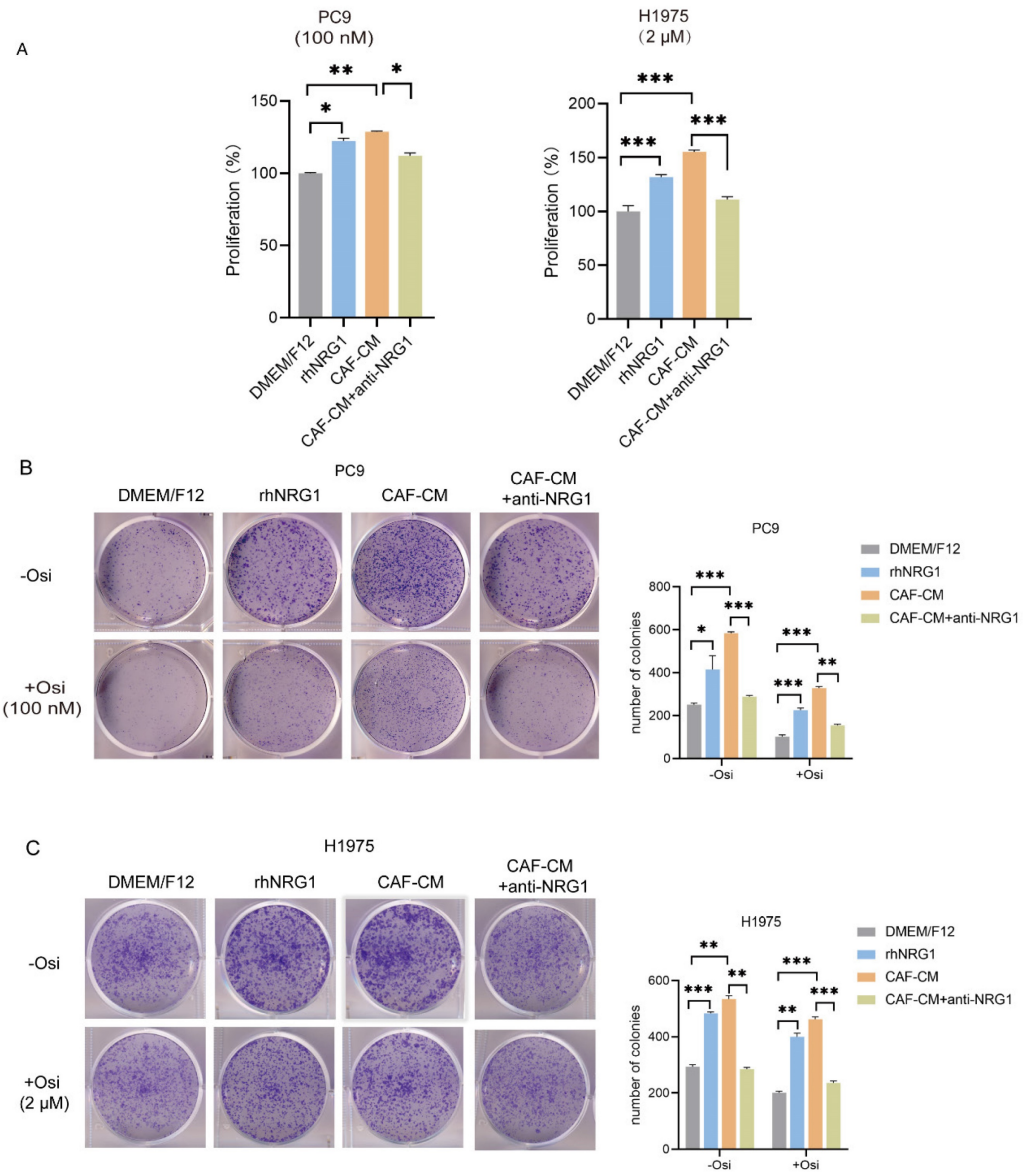

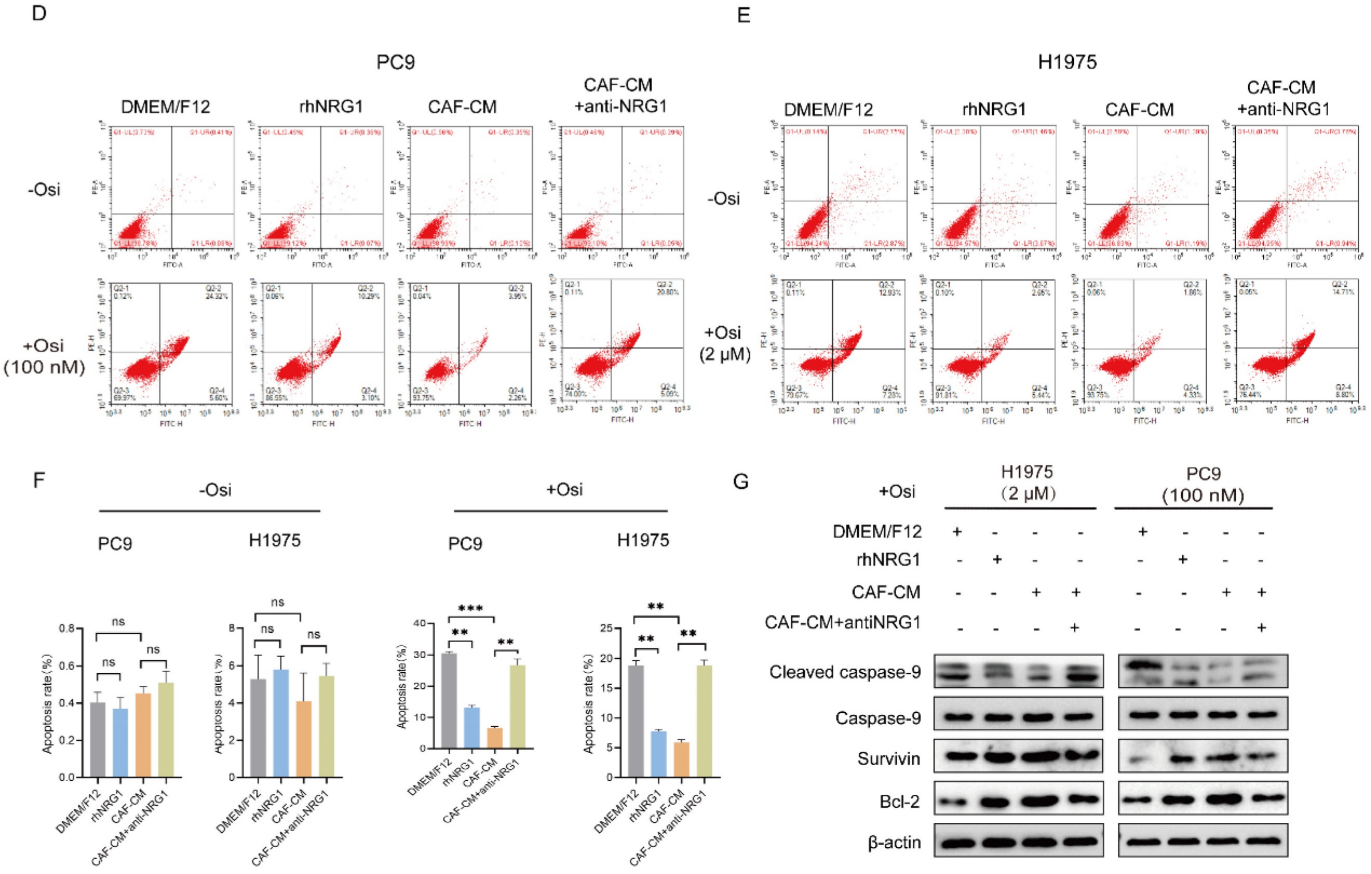

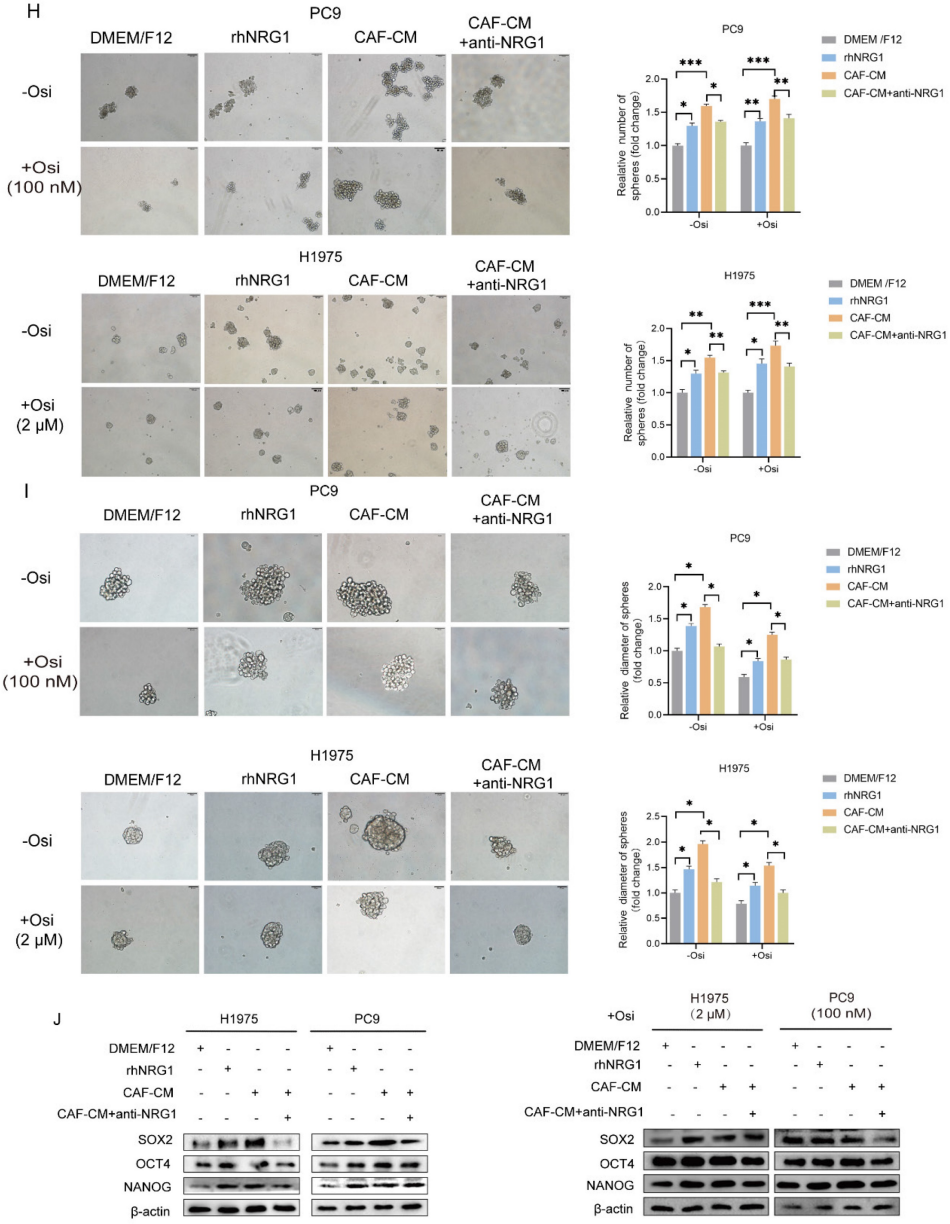
** NRG1 mediates the effect of CAFs on resistance of NSCLC cell to osimertinib.** NSCLC cells were cultured in DMEM/F12 with rhNRG1 (100 ng/mL) or CAF-CM with NRG1 neutralizing antibody (100 ng/mL), and treated with osimertinib. (**A**) Cell viability was detected by CCK-8 kit after 48 h. (**B, C**) The colony formation assay was performed after 1-2 weeks. (**D-F**) The apoptosis level of NSCLC cells was detected by flow cytometry after 48 h. (**G**) The protein expressions were detected by Western blotting after 48 h. (**H-I**) Cell sphere formation experiment was performed after 1-2 weeks. H: scale bar = 100 μm. I: scale bar = 50 μm. (**J**) The protein expressions were detected by Western blotting after 48 h. Data represented the mean ± SD from three independent experiments. Columns, mean; bars, SD. *P<0.05, **P<0.01, ***P<0.001.

In order to explore the role of CAF-secreted NRG1 in osimertinib resistance, we applied rhNRG1 or NRG1 neutralizing antibody to the culture medium. First, we examined their effect on cell viability under osimertinib treatment. We found that both rhNRG1and CAFs promoted cell proliferation and osimertinib resistance, interestingly, when NRG1 neutralizing antibody was added to CAF-CM, CAF's effect on lung cancer cell proliferation was abrogated (Figure 5A). Colony formation experiments also indicated that NRG1 and CAFs enhanced the colony-forming ability of NSCLC cells, while blocking NRG1 with neutralizing antibodies attenuated CAFs' enhancing effect, in both untreated- and osimertinib-treated group (Figure 5B, C).

Next, the apoptosis induction was examined. The flow cytometry analysis demonstrated that NRG1 hardly affected the apoptosis rate in untreated group, however, it effectively protected cells from apoptosis induced by osimertinib, meanwhile, NRG1 neutralizing antibody eliminated CAFs' protecting effect (Figure 5D-F). To further confirm this, we examined apoptosis-related genes, and found that NRG1 and CAFs suppressed caspase-9 activation and increased Survivin and Bcl-2 expression, whereas, NRG1 neutralizing antibody abolished CAFs' effect (Figure 5G). Furthermore, the effect of NRG1 on lung cancer cell stemness was evaluated. By applying rhNRG1 and NRG1 neutralizing antibody, both sphere formation assay and stemness-related gene examination revealed that CAFs facilitated lung cancer cell stemness under osimertinib treatment via NRG1 (Figure 5H-J). Collectively, these findings illustrated that NRG1 mediates the effect of CAFs on osimertinib resistance in NSCLC cells.

### CAFs promoted the resistance of NSCLC cells to osimertinib via NRG1-mediated HER3/AKT/NF-κB pathway

**Figure 6:**
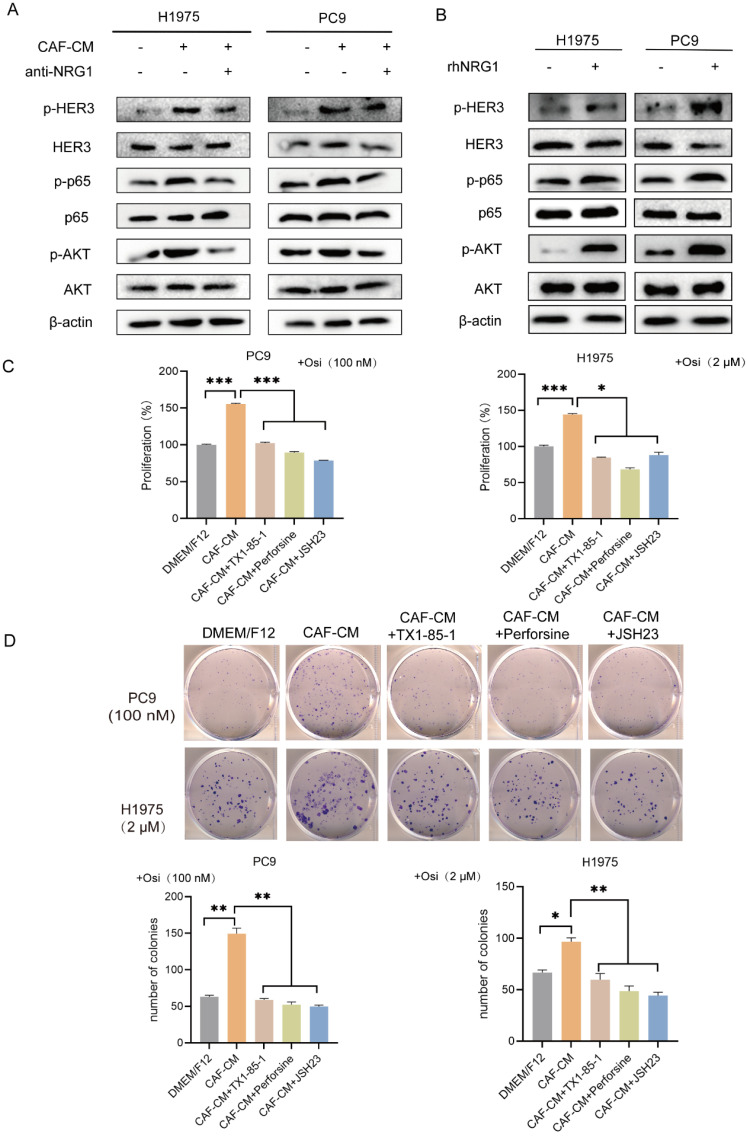
** CAFs promote the resistance of NSCLC cell to osimertinib via NRG1-mediated HER3/AKT/NF-κB pathway.** (**A, B**) NSCLC cells were cultured in DMEM/F12 with rhNRG1 (100 ng/mL) or CAF-CM with NRG1 neutralizing antibody (100 ng/mL). The protein expression and phosphorylation were detected by Western blotting after 48 h. (**C**) NSCLC cells were pre-treated with HER3 inhibitor TX1-85-1(1 μM), AKT inhibitor perifosine (1μM) or NF-κB inhibitor JSH23 (10 μM) for 2 h, then cultured in CAF-CM and treated with osimertinib (100 nm or 2 μM) for 48 h. Cell viability was detected by CCK8. (**D**) NSCLC cells were pre-treated with HER3 inhibitor TX1-85-1(1μM), AKT inhibitor perifosine (1μM) or NF-κB inhibitor JSH23 (10μM) for 2 h, then were cultured in CAF-CM and treated with osimertinib (100 nm or 2 μM). The colony formation assay was performed after 1-2 weeks. Columns, mean; bars, SD. *P<0.05, **P<0.01, ***P<0.001.

NRG1 is an ERBB3 (HER3) ligand. The direct binding of NRG1 to ERBB3 promotes the dimerization of ERBB3 to ERBB2 (HER2), which possesses strong kinase activity. This further activates a variety of signal cascades such as AKT and MAPK signaling pathways 13. Notably, AKT/NF-κB signaling pathway is involved in regulation of tumor progression, especially drug resistance 18, 19, thus providing rationale for the choice of HER3-AKT signaling for further study on osimertinib resistance. We examined the effect of CAFs on the HER3/AKT/NF-κB pathway. As shown in Figure 6A, CAFs elevated the levels of p-HER3, p-AKT and p-p65, meanwhile the levels of total HER3, AKT and p65 remained unchanged. However, when NRG1 neutralizing antibody was added to CAF-CM, the pathway activation was attenuated. We then added rhNRG1 to culture medium, as expected, NRG1 activated HER3/AKT/NF-κB pathway effectively, this result further demonstrated that CAFs activated HER3/AKT signaling via NRG1 (Figure 6B).

Furthermore, we investigated whether HER3/AKT/NF-κB signaling pathway is responsible for CAFs' effect on osimertinib resistance. NSCLC cells were pretreated with HER3 selective inhibitor TX1-85-1, AKT selective inhibitor perifosine or NF-κB selective inhibitor JSH23, then treated with osimertinib. Both cell proliferation assay and colony formation assay revealed that inhibition of HER3/AKT/NF-κB pathway dramatically blunted the enhancing effect of CAFs on osimertinib resistance (Figure 6C, D). Taken together, our results indicated that CAFs promoted the resistance of NSCLC cell to osimertinib via NRG1-mediated HER3/AKT/NF-κB pathway.

### CAFs enhance osimertinib resistance of NSCLC cell *in vivo*

**Figure 7:**
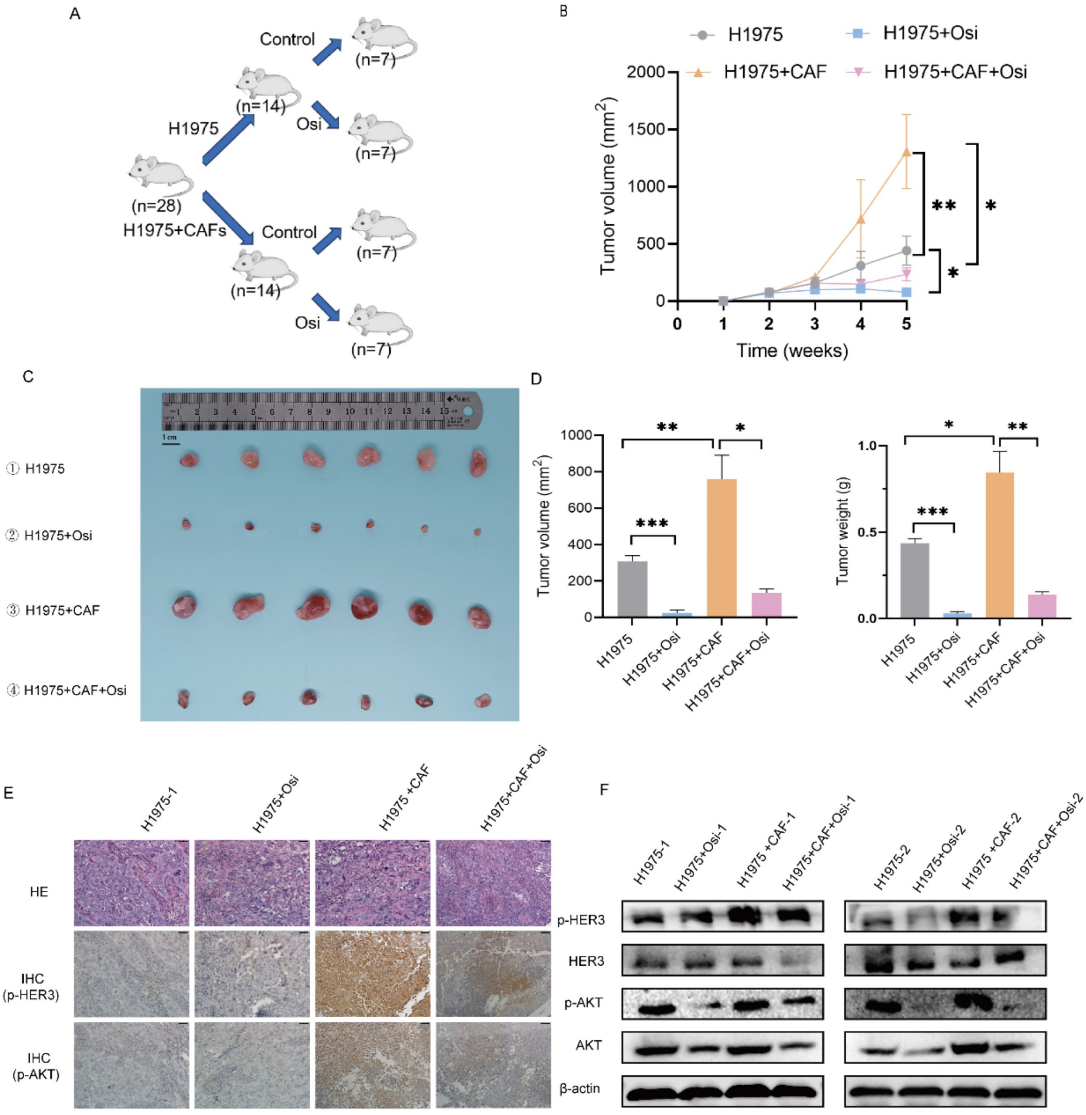
** CAFs facilitate osimertinib resistance of NSCLC cells *in vivo.*** 1×10^6^ H1975 cells, alone or with 3×10^6^ CAFs (ratio1:3) were injected subcutaneously into BALB/c nude mice. Mice were treated with deionized water or osimertinib (2.5 mg/kg body weight) for 5 weeks. (**A, B**) Tumor growth was observed and measured every week. (**C, D**) When experiment was terminated, tumors were excised, and tumor volume and weight were measured. Scale bar = 1 cm. (**E**) The p-HER3 and p-AKT levels in tumor tissues were detected by IHC assay. (**F**) The p-HER3 and p-AKT levels in tumor tissues were detected by Western blotting. Columns, mean; bars, SD. **p < 0.01, ***p < 0.001.

In order to explore the effect of CAFs on osimertinib resistance *in vivo*, we performed animal experiments to evaluate the effect of CAFs on tumor growth and drug resistance by using xenograft tumor model. The mice were divided into four treatment groups: (1) H1975 cells; (2) H1975 cells treated with osimertinib; (3) H1975 cells plus CAFs; (4) H1975 cells plus CAFs treated with osimertinib. The tumor growth was assessed by measuring tumor volume and tumor weight. As shown in Figure 7A-C, CAFs accelerated tumor growth dramatically. Notably, although osimertinib inhibited tumor growth effectively, CAFs enhanced osimertinib-treated tumor growth.

Moreover, we investigated the underlying mechanism by examining tumor tissues. Immunohistochemistry assay indicated that CAFs increased the level of p-HER3 and p-AKT in tumor tissues, Western blotting analysis further verified the elevated phosphorylation level of HER3 and AKT (Figure 7D, E). These findings were in line with our *in vitro* study, indicating that CAFs facilitated osimertinib resistance of lung cancer cells through HER3-AKT pathway.

## Discussion

Over decades the study on cancer drug resistance has been mainly focused on cancer cells, until recent years it has been revealed that cancer progression depends on the crosstalk between cancer cells and TME, and TME plays a crucial role in drug resistance. CAFs are major stromal cells in TME, they promote drug resistance in various types of cancer. Our previous study showed that CAFs potentiated cisplatin resistance of lung cancer cells. Further mechanism study illustrated that CAFs decreased the chemo-sensitivity of lung cancer cells through inhibiting cisplatin-induced apoptosis by activating ANXA3/JNK signaling pathway 10.

Targeted therapy has achieved significant clinical outcomes in cancer treatment; however, the acquired resistance remains unsolved. To date, there are limited studies reporting the role of CAFs in the resistance of cancer cell to targeted therapy. CAFs promote resistance of renal cell carcinoma to sunitinib via secreting CXCL3 and activating ERK1/2 signaling pathway and EMT 20. CAFs decreased the sensitivity of lung cancer cells to anlotinib by reducing apoptosis rate 21. Wei G *et al* reported that CAFs regulate HK2-mediated glycolysis through SKP2, promoting almonertinib resistance in NSCLC 22.

Notably, CAFs are also responsible for osimertinib resistance. CAFs contribute to osimertinib resistance in lung cancer cells via promoting ribosome biosynthesis 23, they promote osimertinib resistance and lung cancer progression via periostin-mediated ERK activation and EMT 24. Interestingly, Zhu K *et al* reported that CAFs enhance osimertinib resistance via MET/AKT/Snail signaling pathway, and inhibition of this pathway may overcome osimertinib resistance by targeting both CAFs and cancer cells 25. In our study, we found that CAFs facilitated osimertinib resistance through promoting cell growth and colony formation, protecting lung cancer cells from apoptosis, and enhancing stemness of lung cancer cells.

The stemness of cancer cell is critical in drug resistance. Several studies have shown that stemness of cancer cell also play an important role in osimertinib resistance. P21-activated kinase 2 (PAK2) - mediated activation of β-catenin pathway enhances lung cancer cell stemness and osimertinib resistance 26. miR-204 represses lung cancer cell stemness and promotes osimertinib sensitivity by targeting CD44 27. SHP2 inhibition boosts the anticancer effect of osimertinib by blocking CXCL8 loop-mediated stemness 28. Accumulating evidence indicates that CAFs contribute to drug resistance via regulating cancer cell stemness. CAFs enhance stemness and gemcitabine resistance via HIF-1alpha/miR-21 axis in pancreatic cancer 8. CAFs facilitates colorectal cancer stemness and oxaliplatin resistance of colorectal cancer cells through transcriptionally activating ITGB4 29. Ma Y *et al* reported that downregulation of SPARC in CAFs stimulates stemness transformation and 5-fluorouracil resistance in gastric cancer 30. However, the role of cell stemness in CAFs-mediated osimertinib resistance is unclear. In this study, we demonstrated that CAFs enhance osimertinib resistance via regulating cell stemness.

The interaction between CAFs and tumor cells is essential for tumor development. CAFs facilitate tumor progression via secreting cytokines and growth factors, and various mechanisms are involved. CAFs promote hepatocellular carcinoma malignancy by SERPINH1 secretion and regulating SENP3-mediated SP1/SQLE pathway 31. CAFs promote oral squamous cell carcinoma progression by targeting ATP7A through exosome-mediated paracrine miR-148b-3p 32. Zhang K *et al* reported that CAFs promote doxorubicin resistance in triple-negative breast cancer by regulating ferroptosis through the enhancement of ZFP64 histone lactylation 33. In our previous studies, we demonstrated that CAFs potentiate metastasis and chemoresistance of lung cancer cells via secreting IL-6, ANXA3, VEGFA, HMGB1 10, 15, 34, 35. When compared the differentially expressed genes between CAFs and NFs by RNA sequencing 10, we found that NRG1 is highly expressed in CAFs, thus provided rationale for further explored the role of NRG1 in CAFs' effect on osimertinib resistance.

NRG1 is a prognostic biomarker in glioblastoma, it promotes malignancy by inhibiting autophagy via AKT/mTOR pathway 36. NRG1 regulates metastasis of triple-negative breast cancer cells via c-myc ubiquitination 37. Hou G* et al* reported that NRG1 stimulates tumorigenesis and metastasis of esophageal squamous cell carcinoma 38. Interestingly, NRG1 also contributes to CAFs' effect on drug resistance. The expression level of NRG1 is higher in CAFs than in HER2-positive breast cancer cells, and NRG1 mediated the resistance of breast cancer cells to trastuzumab 39. In prostate cancer, CAFs-derived NRG1 promotes antiandrogen resistance through HER3 activation 40. However, it has not been evaluated whether NRG1 is involved in osimertinib resistance. In the present study, we demonstrated that lung cancer derived-CAFs possessed a high level of NRG1, and CAFs-secreted NRG1 mediated the effect of CAFs on resistance of lung cancer cell to osimertinib. Interestingly, we found that osimertinib stimulated NRG1 secretion from CAFs, which may further enhance the osimertinib resistance.

NRG1 is an ERBB3 (HER3) ligand, it directly binds to ERBB3 to promote the dimerization of ERBB3 to ERBB2 (HER2). This further activates a variety of signal cascades such as AKT signaling pathway 13. In order to further elucidate the underlying mechanism, we investigated the downstream signaling pathways. By using the selective inhibitors of HER3, AKT and NF-κB, we demonstrated that CAFs-derived NRG1 activated HER3/AKT/NF-κB pathway to facilitate osimertinib resistance.

**Figure 8:**
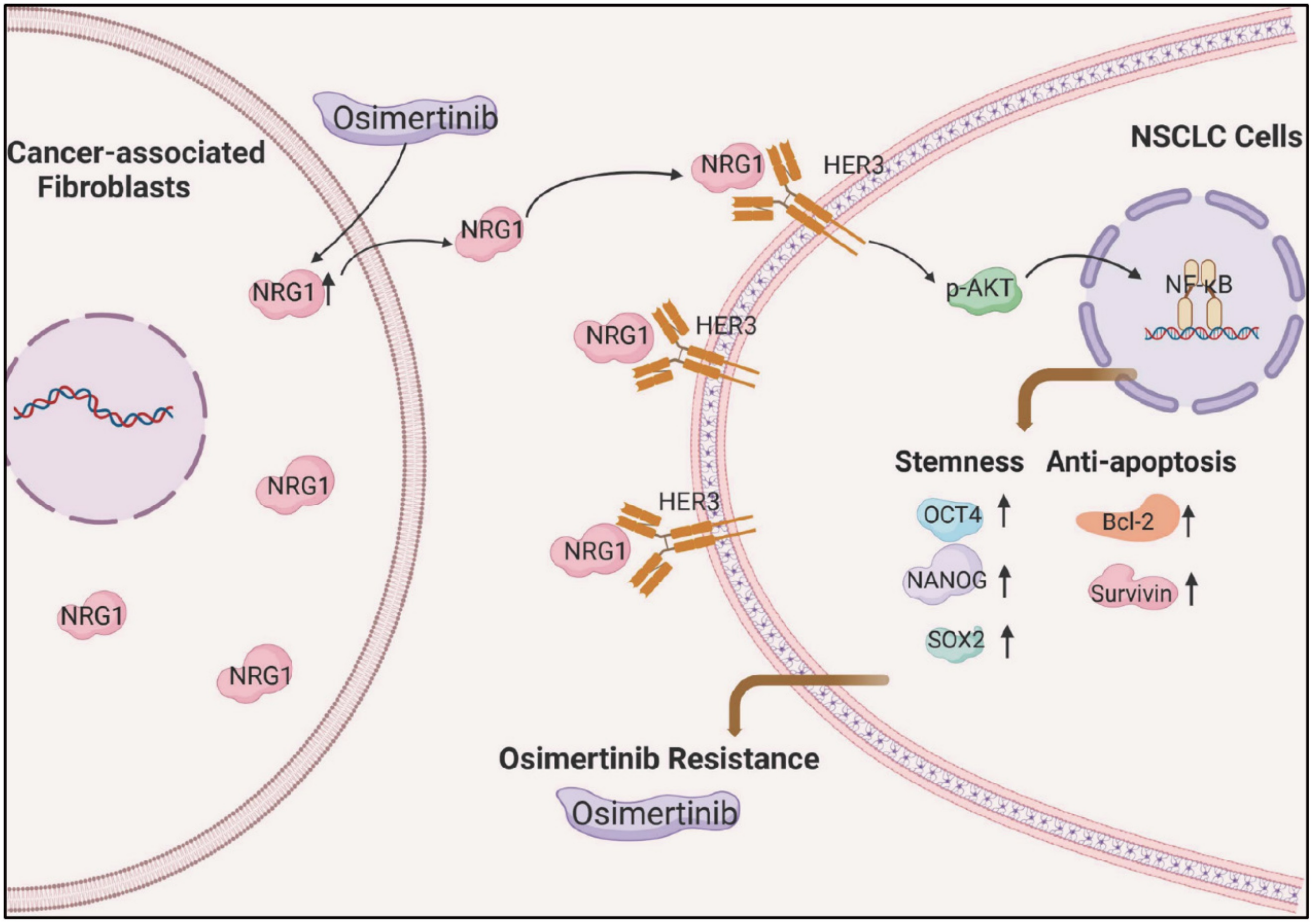
Scheme illustrating the promoting effect of CAFs on osimertinib resistance in non-small cell lung cancer cells.

In summary, we demonstrated that CAFs promotes osimertinib resistance of lung cancer cells via NRG1-mediated activation of HER3/AKT/NF-κB signaling pathway (Figure 8). Meanwhile, osimertinib stimulates NRG1 secretion from CAFs, which may further facilitate the osimertinib resistance. Our finding suggests the potential value of CAFs-derived NRG1 as a novel therapeutic target for osimertinib resistance in lung cancer (Figure 8). It is worth to note that subpopulations of CAFs have been identified in several types of cancer 41-43, in particular, in lung cancer 44-46. Further evaluation of the role of CAFs subpopulations in drug resistance will provide useful information for precise targeted therapy.
